# A robust and parsimonious machine learning method to predict ICU admission of COVID-19 patients

**DOI:** 10.1007/s11517-022-02543-x

**Published:** 2022-03-30

**Authors:** Lorenzo Famiglini, Andrea Campagner, Anna Carobene, Federico Cabitza

**Affiliations:** 1 Department of Informatics, University of Milano-Bicocca, Milan, Italy; 2 IRCCS San Raffaele Scientific Institute, Milan, Italy; 3 IRCCS Orthopedic Institute Galeazzi, Milan, Italy

**Keywords:** COVID-19, Prognostic models, Machine learning, eXplainable AI, Complete blood count

## Abstract

**Abstract:**

In this article, we discuss the development of prognostic machine learning (ML) models for COVID-19 progression, by focusing on the task of predicting ICU admission within (any of) the next 5 days. On the basis of 6,625 complete blood count (CBC) tests from 1,004 patients, of which 18% were admitted to intensive care unit (ICU), we created four ML models, by adopting a robust development procedure which was designed to minimize risks of bias and over-fitting, according to reference guidelines. The best model, a support vector machine, had an AUC of .85, a Brier score of .14, and a standardized net benefit of .69: these scores indicate that the model performed well over a variety of prediction criteria. We also conducted an interpretability study to back up our findings, showing that the data on which the developed model is based is consistent with the current medical literature. This also demonstrates that CBC data and ML methods can be used to predict COVID-19 patients’ ICU admission at a relatively low cost: in particular, since CBC data can be quickly obtained by means of routine blood exams, our models could be used in resource-constrained settings and provide health practitioners with rapid and reliable indications.

**Graphical abstract:**

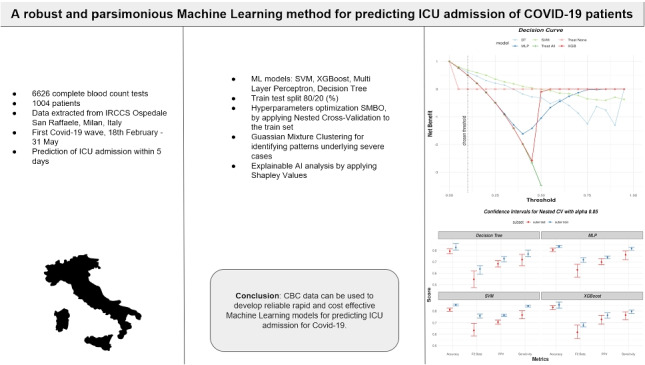

## Introduction

The coronavirus SARS-CoV-2 has infected over 300 million individuals and killed almost six million people in the first 2 years since it first appeared. Artificial intelligence approaches supporting different healthcare activities have drawn increasing interest as a way to curb the spread of this unprecedented pandemic. Nonetheless, the development of prognostic models, either to predict ICU admission or other outcomes, or to stratify patients according to risk, has yet to yield clearly useful results: recent studies report significant limitations (in terms of bias or overfitting) in existing solutions [2, 3, 35].

To address and overcome these shortcomings, we report the results of a retrospective study in which we developed some machine learning (ML) prognostic models to predict ICU admission, which may be viewed as a sign of illness severity. Our study, which is a follow-up to a previous successful pilot [11], grounds on one of the most reliable datasets for COVID-19 that is freely available (on Zenodo https://zenodo.org/record/4081318#.X_1UDxYo-Uk) for analysis to date, as it was directly curated by one of the most important Italian laboratories. Due to its widespread use in various diagnostic and monitoring activities, from this dataset, we extracted a limited collection of features of standard blood tests that are both affordable and easy to execute, the so-called complete blood count (or CBC). This choice is supported by the vast literature showing important correlations between these parameters and the COVID-19 course and prognosis [12, 23].

We considered various ML algorithms, and adopted a development pipeline aimed at minimizing data leakage and the risks of bias and overfitting. A support vector machine (SVM) model showed very good performance, in terms of discrimination, calibration, and utility. Furthermore, an explainable AI study backs up the model comprehensibility, showing its potential to help clinicians make better decisions during the care and treatment of COVID-19 patients by providing them with interpretable clues. To our knowledge, this is the first study to use ML methods to predict COVID-19 ICU admission based only on CBC values while adhering to rigorous reporting and replicability standards [4, 19].

## Methods

The study protocol (BIGDATA-COVID19) was approved by the Institutional Ethical Review Board in agreement with the World Medical Association Declaration of Helsinki. In what follows, we report the ML methodology, as well as the results, based on the IJMEDI checklist [4] for the reporting and evaluation of ML studies.

### Data understanding

The dataset used for this retrospective study encompasses the results of routine blood tests of 1,361 patients, regularly admitted to the hospital emergency department for COVID-19 of the San Raffaele Hospital (OSR), Milan (Italy). The data collection was performed between February 19, and May 31, 2020, i.e., at the height of the first wave of the epidemic in Italy. All patients admitted to the ED in the time period were initially included for analysis.

The data, which was manually extracted from the OSR laboratory system by one of the authors, represent the entire population of patients admitted into the emergency department (ED) of the hospital (which was COVID center in the Milan area) during the first wave of the pandemic and, therefore, we assume it is representative of the COVID-19 population at that time in that catchment area.

For each patient who stayed at the hospital for at least 24 h, multiple observations were considered (approximately one for each day of hospital stay), each one corresponding to a single time window.

As covariate features, we selected a set of 22 variables: namely gender, age, and the complete blood count (CBC), including the leukocyte formula (analyzed through a Sysmex XN 9000 haematology analyzer).

**Fig. 1:**
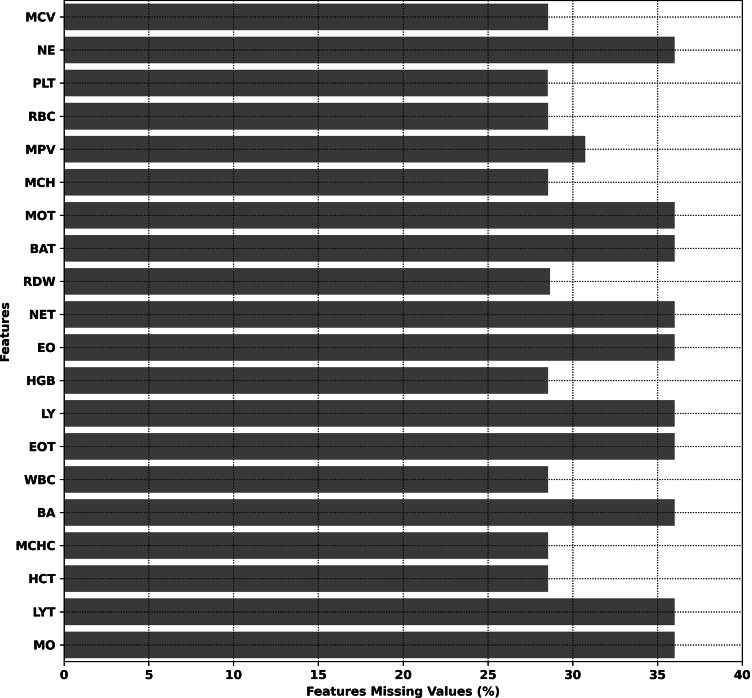
Percentage of missing values in the dataset

In total, the dataset extracted from the EHR encompassed 11,103 unique records for 1,361 unique patients. The dataset is publicly available on Zenodo^1^. The distribution of missing values in the dataset is reported in Fig. 1.

In order to avoid excessive bias due to imputation procedures, records with more than 30% of missing feature values were excluded from further analysis. As a result, a total of 6,625 records associated with 1,004 unique patients (5.8 records for patient on average, largest number of records 31) were included in the study.

In regard to the target (i.e., admission to the ICU, as an indicator of prognostic worsening), we considered the event that the patients entered in the ICU within the following 5 days: that is, for each record (corresponding to a day *d* of hospital stay, for a given patient *p*), the target variable was set to 1 if the patient *p* under consideration was admitted in ICU within days *d* and $d+5$. Otherwise, the target variable was set to 0.

The descriptive statistics for the included patients are reported in Table 1. A total of 4,492 records were associated with male patients (502 patients), while 2,133 records were associated with female patients (502 patients). The mean time of hospitalization for men was 10.7 days, while for women it was 7.9 days. 78% of the patients who had to be transferred to the ICU within 5 days were men, while 22% were women. In particular, out of the total number of men, 7% were admitted to the ICU, while out of the total number of women, approximately 3% were admitted to the ICU. The maximum time span from admission to discharge was 31 days. In regard to the age distribution, 1.23% of the patients ($N=12$) were aged less than or equal to 18 years, while the remaining 98.77% of the patients were of majority age. The dataset was strongly imbalanced: only 18.3% of the records were associated with ICU admission.

**Table 1:** Distribution of the demographic and CBC predictive features

Feature	Unit of measure	Mean	Std	Min-max range	25–75%	Missing (%)
Mean corpuscular volume (MCV)	$10^9/L$	88.26	6.90	[54.7, 121.5]	[85, 92.4]	0
Neutrophils count (NE)	%	70.69	14.97	[9.4, 99.6]	[61.2, 82.4]	0
Platelets (PLT)	$10^9/L$	269.41	125.44	[10, 1019.5]	[180, 337]	0
Red blood cells (RBC)	$10^{12}/L$	4.21	0.75	[1.75, 7.12]	[3.7, 4.73]	0
Mean platelet volume (MPV)	fL	10.74	1.07	[7.8, 15.6]	[10, 11.32]	3.03
Mean corpuscular hemoglobin (MCH)	pg/Cell	29.09	2.60	[15, 65.7]	[28.1, 30.5]	0
Monocytes count (MOT)	$10^9/L$	0.67	0.38	[0, 4.8]	[0.4, 0.9]	0
Basophils count (BAT)	$10^9/L$	0.03	0.05	[0, 0.7]	[0, 0]	0
Erythrocyte distribution width (RDW)	CV%	14.36	2.36	[6.71, 31.8]	[12.9, 15.1]	0.16
Neutrophils count (NET)	$10^9/L$	6.63	4.58	[0.3, 47.4]	[3.5, 8.4]	0
Eosinophils count (EO)	%	1.83	2.50	[0, 34.8]	[0.1, 2.5]	0
Hemoglobin (HGB)	g/dL	12.20	2.11	[4.8, 19.7]	[10.6, 13.7]	0
Lymphocytes count (LY)	%	18.69	12.01	[0.1, 76]	[9.4, 25.3]	0
Eosinophils count (EOT)	$10^9/L$	0.15	0.28	[0, 5]	[0, 0.2]	0
White blood cells (WBC)	$10^9/L$	8.89	5.15	[0.7, 111.1]	[5.6, 10.9]	0
Basophils count (BA)	%	0.44	0.35	[0, 3.4]	[0.2, 0.6]	0
Mean corpuscular hemoglobin concentration (MCHC)	g Hb/dL	32.96	1.47	[25.9, 58.7]	[32.1, 33.9]	0
Hematocrit (HCT)	%	36.95	5.91	[16.8, 59.7]	[32.7, 41.1]	0
Lymphocytes count (LYT)	$10^9/L$	1.40	1.55	[0, 82.8]	[0.8, 1.7]	0
Monocytes count (MO)	%	8.35	3.93	[0, 38.9]	[5.5, 10.7]	0
Age	Years	64.76	15.47	[0, 100]	[55, 77]	0

**Fig. 2:**
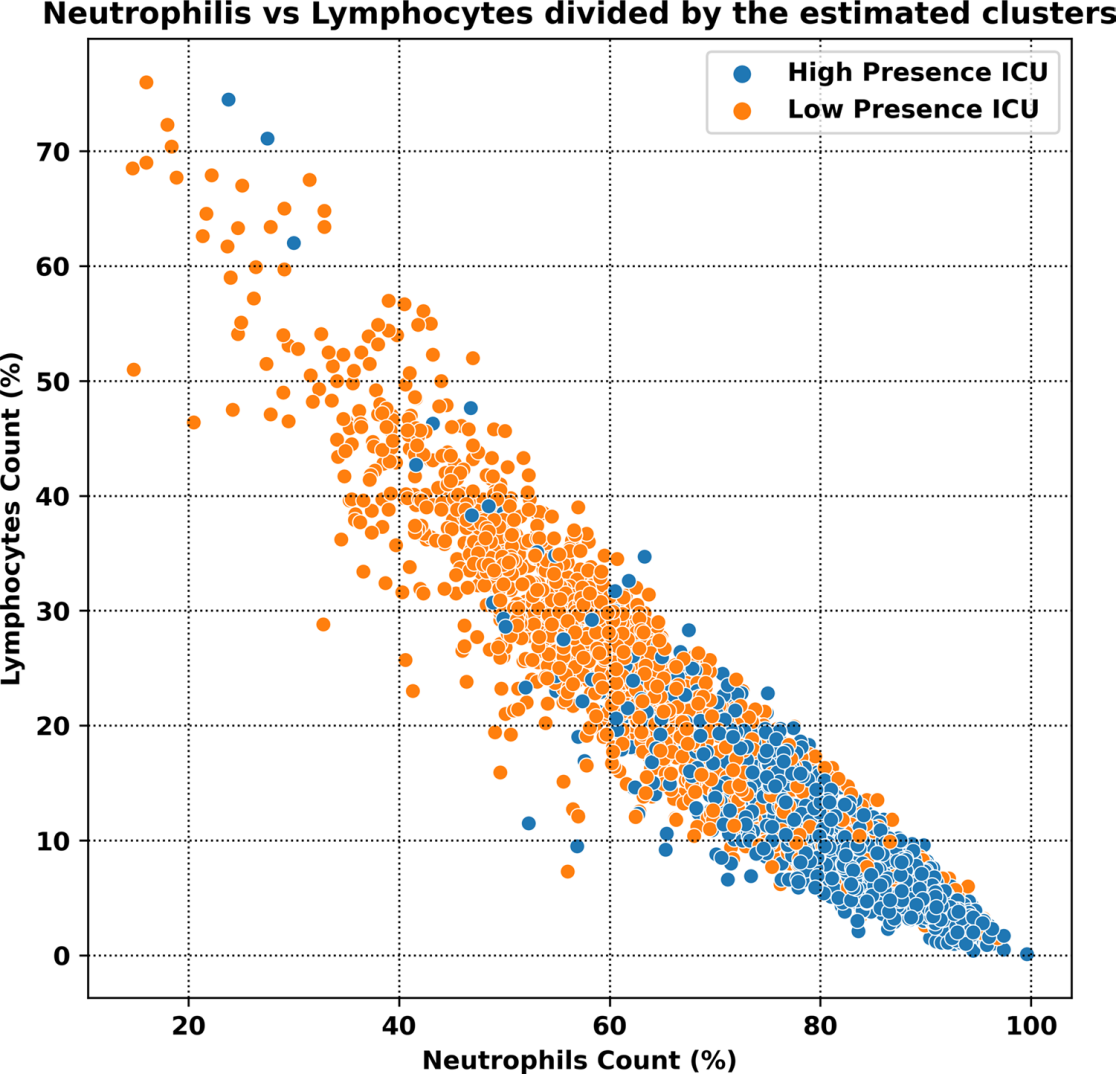
Scatter plot of the lymphocytes and neutrophils counts for the patients in the severity and normal clusters (see Section 2.2)

In order to assess the presence of significant differences among the records associated with ICU admission and the other records, we applied a descriptive clustering approach. Namely, for each time window (thus, between 0, i.e., admission date, and 31), we performed clustering using 2 clusters. The Gaussian mixture algorithm was selected for this purpose. Prior to clustering, the dataset was normalized by applying record-wise L2 normalization, so as to re-scale the different subsets of the data independently. Then, after applying clustering to each time window, we compared the percentage of patients admitted to the ICU in the two clusters. Fisher’s exact test was applied for this comparison, using a confidence level equal to 95% (i.e., $\alpha = 0.05$). For each time step, we also computed the Silhouette index as a measure of clustering fitness (i.e., the higher the Silhouette index, the better the obtained clustering). The results of the clustering analysis are reported in Table 2. Furthermore, in Fig. 2, we report the distribution of two features (which have been shown to be strongly correlated with COVID-19 prognosis [16, 29]) within the two clusters. As shown in Table 2, for the first 10 time windows, most of the comparisons (7 out of 10) reported a significant difference among the induced clusters, while the significance of the tests decreased when increasing the length of hospital stay, due to small sample sizes. In particular, for the last 3 time windows, the obtained clusterings were not sufficiently well separated (one of the two clusters encompassed a single record). Due to these positive results, the information about clustering was included as an additional feature for the development of classification models (see Section 2.2).

**Table 2:** Results of the Silhouette index and Fisher exact test for comparing the proportion of ICU admissions in the two clusters, for each time window

Time window	*p* value	Silh
0	0.0002	0.75
1	0.0166	0.72
2	0.0674	0.73
3	0.0205	0.72
4	0.0235	0.74
5	0.0215	0.73
6	0.0645	0.74
7	0.0582	0.73
8	0.0494	0.74
9	0.0121	0.73
10	0.1118	0.75
11	0.2558	0.75
12	0.2077	0.74
13	0.4504	0.75
14	0.2454	0.75
15	0.1161	0.74
16	0.3942	0.75
17	0.0782	0.74
18	0.0515	0.73
19	1	0.72
20	0.0864	0.72
21	0.2182	0.73
22	0.4357	0.71
23	0.2728	0.70
24	0.0872	0.74
25	0.5271	0.72
26	0.5294	0.73
27	0.3016	0.75
28	0.6631	0.71
29	NA	0.74
30	NA	0.74
31	NA	0.76

### Data preparation

As an initial step before development of the machine learning models, the complete dataset was randomly split into a training set (encompassing 80% of the records) and a test set (encompassing the remaining 20% of the records). The data split was performed so that all records pertaining to any given patient were in the same split, to avoid data leakage. As described below (see Section 2.3), training and hyper-parameter optimization of the models were performed on the training set (based on a nested cross-validation procedure), while the final evaluation was performed on the separate hold-out test set.

Since the dataset was affected by missing data, imputation was applied, as a preliminary step to model development. Imputation was performed by means of an iterative multivariate imputation approach, based on the Bayesian ridge estimator, in order to better account for potential correlations in the distribution of the features. While imputation was performed contextually to model training (see Section 2.3), in order to avoid data leakage, we first assessed the potential bias induced by the imputation procedure. To do so, we compared the distributions of the features before imputation and after imputation, for 50 different random imputations, in order to assess for any statistically significant difference among the distribution of the features. The comparison was performed by means of the Kolmogorov-Smirnov test, for each feature, using a confidence level of 95% (i.e., $\alpha = 0.05$). The null hypothesis (i.e., the two distributions are not significantly different) was considered as a proxy for the absence of imputation-induced bias. In order to correct for multiple comparisons, the Benjamini-Hochberg procedure was applied. All comparisons were associated with a *p*-value greater than 0.05. Thus, the null hypothesis could not be rejected, and the imputation procedure was considered unbiased.

As mentioned in the previous section, based on the results of the descriptive clustering analysis, we created an additional categorical variable with the following classes: *low presence ICU*, *high presence ICU*, *not significant*, and *low sample*. In order to avoid data leakage, the assignment of these categories was performed based on the training set. In particular, the clustering procedure was repeated on the training set to obtain a clustering for each time window. Based on the results of Fisher’s exact test, the *low presence ICU* and *high presence ICU* categories were created when, in a given time window, there was a significant difference among the two clusters. In particular, the cluster with the highest percentage of ICU admissions was assigned the *high presence ICU* label. As for the category *not significant*, it denotes the fact that the difference between the two clusters was not significant. Finally, the category *low sample* was assigned to the records in the time windows 29, 30, and 31. The category assignments in the test set were then determined based on the distance between each instance in the latter and the centroids of the clusters as identified in the training set. Together with the time window, the clustering category was used as an additional feature for developing the ML models.

### Modeling

In regard to model development, we considered 4 different families of ML models, namely decision tree (DT), XGBoost (XGB), multi-layer perceptron (ML), and support vector machine (SVM).

As mentioned in the previous sections, the training and hyper-parameter selection of the ML algorithms were performed on the training set, based on a nested k-fold cross-validation (CV) procedure with 7 folds for the outer CV and 5 folds for the inner CV. In particular, for each iteration of the outer CV, the dataset was split in 6 folds (i.e., 86% of the training set, 69% of the complete dataset) used for training and hyper-parameter selection, and 1 fold (i.e., 14% of the training set, 11% of the complete dataset) used for testing. For each iteration of the inner CV, on the other hand, the considered part of the dataset was split in 4 folds used for training (i.e., 69% of the training set, 55% of the complete dataset) and 1 fold used for hyper-parameter selection (i.e. 17% of the training set, 14% of the complete dataset). For both the outer and inner CV, the split was performed so as to guarantee that all records pertaining to any given patients were assigned to the same fold to avoid data leakage.

The training procedure encompassed an imputation step, a standardization step (for all models except XGBoost), and an imbalance-correction step. In particular, the imputation model (as previously mentioned, an iterative multi-variate procedure based on the Bayesian ridge estimator) was trained together with the classification models within the nested CV procedure: the parameters of the imputation models were estimated on the training folds (of the inner CV) and applied on the remaining folds. Regarding the imbalance-correction step, the SMOTE algorithm was applied on the training folds of the nested CV procedure and during the final training of the models.

Hyper-parameter optimization was performed through a randomized model-based search procedure using the Optuna framework [1], assigning a budget of 100 evaluations to each model and optimizing for F2 score (in order to weigh more the under-represented positive class). The full range of evaluated hyper-parameter settings is reported in Table 3.

**Table 3:** Range of evaluated hyper-parameters for the ML models. The values selected as a result of the nested cross-validation procedure are highlighted in bold

Algorithm	Hyper-parameter	Value range
DT	criterion	gini, **entropy**
	max_depth	[3, 6] (**4**)
	min_samples_split	[2, 40] (**31**)
	min_samples_leaf	[1, 20] (**20**)
MLP	learning_rate_init	[1e-5, 5e-2] (**0.047**)
	learning_rate	constant, **invscaling**, adaptive
	solver	adam, **sgd**
	max_iter	[5, 100] (**100**)
	first_layer	[10, 150] (**111**)
	second_layer	[5, 100] (**27**)
	alpha	[1e-5, 5e-2] (**0.03**)
XGB	n_estimators	[5, 200] (**32**)
	max_depth	[2, 30] (**2**)
	reg_alpha	[0, 5] (**0**)
	reg_lambda	[0,5] (**2**)
	gamma	[0, 5] (**2**)
	learning_rate	[0.005, 0.5] (**0.0067**)
SVM	C	[1e-10, 1e10] (**0.055**)
	kernel	sigmoid, **rbf**

The nested CV procedure was also used to assess the stability of the ML algorithms. To this purpose, we evaluated the average and standard deviation of the performances (according to different metrics, namely accuracy, sensitivity, specificity, AUC, and F2 score) of the algorithms, on both the training and test folds of the outer CV. In particular, the width of the 95% confidence intervals (centred around the average) was considered as a measure of stability (i.e., the smaller the C.I., the higher the stability). Confidence intervals were computed by means of the bootstrap with 50 re-samplings. After the nested CV procedure, the models were re-trained (using the hyper-parameters selected during the nested CV) and calibrated (using isotonic regression) on the whole training set. The final models were then evaluated on the separate test set in terms of different metrics, namely accuracy, sensitivity, specificity, precision, AUC, and F2 score. These are defined as:

1 $$accuracy=\frac{TP+TN}{TP+TN+FP+FN}$$

2 $$sensitivity=\frac{TP}{TP+FN}$$

3 $$specificity=\frac{TN}{TN+FP}$$

4 $$precision=\frac{TP}{TP+FP}$$

5 $$F2=5\frac{precision\cdot sensitivity}{4\cdot precision+sensitivity}$$

where TP, FP, TN, and FN are, respectively, the number of true positives, false positives, true negatives, and false negatives.

The models were also evaluated in terms of their calibration (through the Brier score and calibration curves) as well as their utility (through the standardized net benefit (sNB) and decision curves). The Brier score and sNB are respectively defined as:

6 $$Brier=\frac{1}{n}\sum_{i=1}^n(y_i-h(x_i))^2$$

7 $$sNB=\frac{TP_\tau}n-\frac{1-\pi}\pi\frac{\tau}{1-\tau}\frac{FP_\tau}n$$

where *n* is the number of cases, $$(x_i, y_i)$$ represents the features and label for case *i*, $$h(x_i)$$ is the confidence score associated to the positive class (i.e., ICU admission) for case *i*, $$\pi = \frac{TP + FN}{n}$$, and $$\tau \in [0,1]$$ is a probability threshold representing the relative cost of false-positive predictions. In regard to this latter parameter, we considered the cost of ICU admission to be $100000 (i.e., the daily cost for ICU patients, as estimated by FAIR Health [10]), and the benefit for the same prediction to be $900000 (i.e., the estimated value of life for a person with a remaining life-span of 18 QALY [20], based on the average life-span in Italy at the time of publication and the average age of the patients involved in the study). The resulting threshold value was $$\tau = 0.1$$.

## Results

**Fig. 3:**
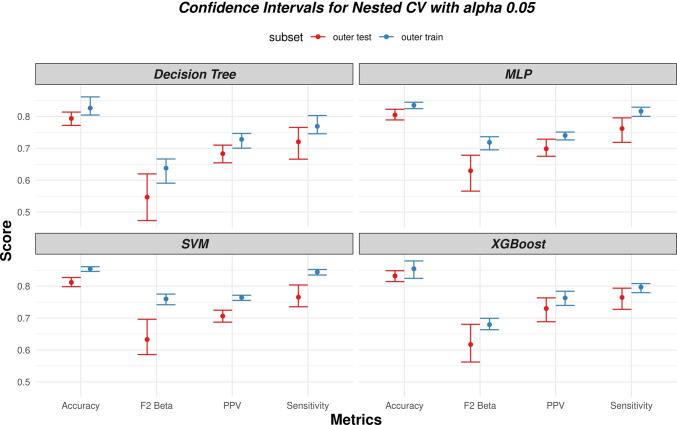
Results of the nested CV for all the evaluated models

The results of the nested CV procedure are reported in Fig. 3. The selected hyper-parameter values are reported in Table 3, in bold.

**Fig. 4:**
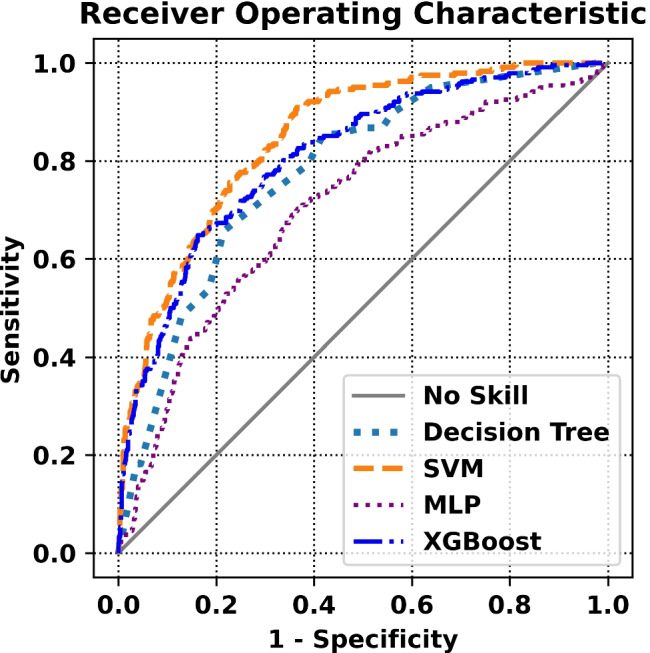
ROC curves for the evaluated ML models

**Fig. 5:**
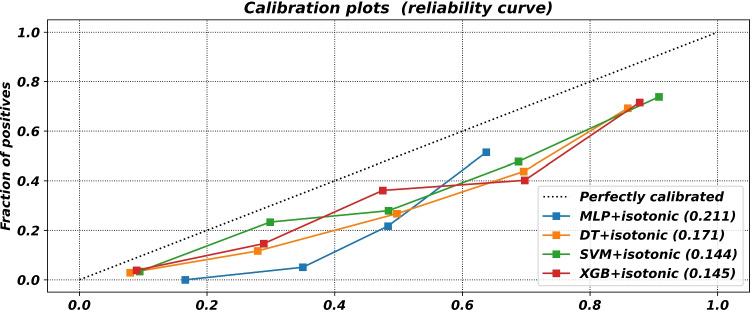
Calibration curves for the evaluated ML models

**Fig. 6:**
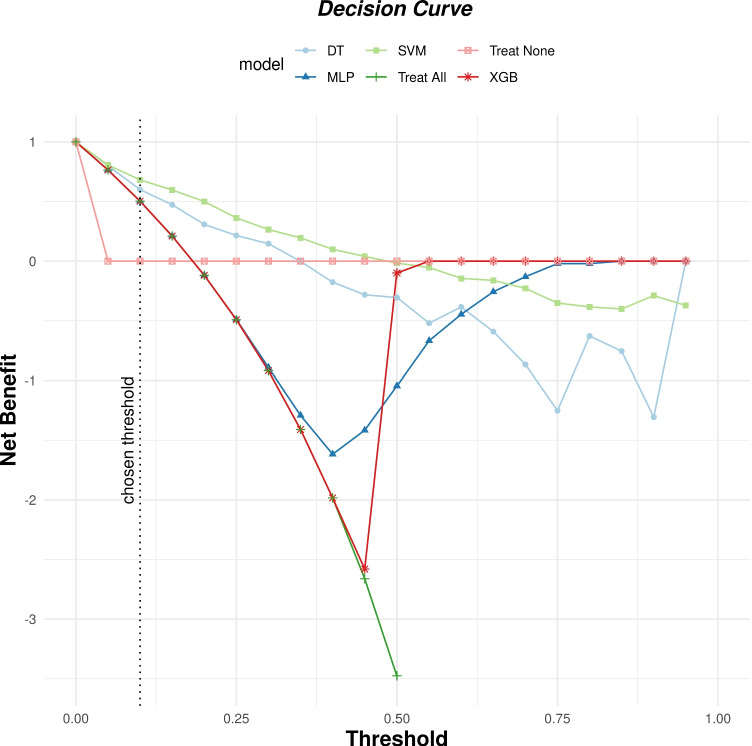
Decision curves for the evaluated ML models

The results of the models on the separate test set are reported in Table 4. Furthermore, the ROC curves, calibration curves, and decision curves for all the evaluated models are reported in Figs. 4, 5, and 6.

**Table 4:** Results of the evaluated ML models

Model	Accuracy	Sensitivity	Specificity	Precision	AUC	F2	Brier	sNB
MLP	0.79	0.29	**0.90**	0.40	0.71	0.31	0.204	0.50
DT	0.76	**0.66**	0.78	0.41	0.76	0.59	0.171	0.60
SVM	**0.82**	0.57	0.88	**0.51**	**0.85**	0.56	**0.144**	**0.69**
XGB	0.80	0.65	0.83	0.46	0.81	**0.60**	0.145	0.50

## Discussion

No wonder that, due to the ongoing impact of the COVID-19 disease on worldwide public health systems, we are not the first researchers to address the problem of ICU admission prediction using machine learning techniques. In what follows, we outline the main characteristics of the most important studies in the existing literature and compare these works with ours in order to highlight the respective strengths and limitations.

Campbell et al. [6] developed a hierarchical risk stratification ensemble model for the prediction of adverse events during hospitalization, including ICU admission, based on a small set of laboratory and clinical features available at admission time. As a consequence of the small data sample used for training (229 patients), the model reported relatively low performance on external validation, with an average sensitivity of 34% and average precision of 64%.

Cheng et al. [7] developed a random forest model based on a multi-modal dataset encompassing laboratory data, clinical characteristics, and ECGs at admission time for 1982 patients. The authors report a sensitivity of 72.8%, a specificity of 76.3%, an accuracy of 76.2%, and an AUC of 79.9%. While these results represent a good trade-off between sensitivity and specificity, the model, as previously mentioned, uses a significant number of characteristics, including laboratory parameters, symptomatology, and electro-cardiological results. This might limit its use in medical practice, particularly in resource-constrained situations. Furthermore, we note that the authors do not provide any detail about the adopted imputation and feature processing methods, thus reducing the study’s replicability.

Fernandes et al. [13] also developed a random forest model for ICU admission, based on laboratory, clinical, and demographics data at admission date for 1040 patients. The model reported an AUC of 96%, a sensitivity of 91%, a specificity of 87%, and a PPV of 72%. While the reported results are higher than those reported by our best model (i.e., SVM), particularly concerning sensitivity, we note that the authors report that feature pre-processing and imputation were performed on the entire dataset before any data split operation. Thus, the reported results may be impacted by data leakage and over-estimation.

Klann et al. [22] developed generalized-linear and gradient-boosting models. They show an average sensitivity of 77% and a specificity of 79% for the task of ICU admission prediction. While the reported results are comparable to ours (and, in particular, the sensitivity is higher), we note that the models use all the information collected in patients’ health records as predictive features, whereas the method we propose only requires CBC data.

Clinical scoring models were developed by Rodriguez-Nava et al. [30] to predict ICU admission, with an average AUC of 76%. Despite this, the scores were produced on a small sample of 300 patients, and they were validated using the same data that was used to develop the score, with no way to prevent overfitting.

Wu et al. [34] created a logistic regression model for risk prediction that was also externally verified. The authors report an AUC of 87%, sensitivity of 86%, and specificity of 71% on the external validation sets. Similarly to the approaches adopted in [7] and [22], the reported model grounds on several features, including hemato-chemical parameters, symptomatology, and radiological results. Finally, while we focus on ICU admission prediction, the authors of the study consider a composite binary prediction task in which a patient is classified as severe if he or she is admitted to the ICU, had organ failure, or died; this, in turn, could further limit the utility of the ML method in the management of severe cases.

Vaid et al. [33] applied different ML models to predict critical events (including ICU admission), based on a dataset encompassing patients’ characteristics and laboratory data at admission time for 4098 COVID-19-positive patients. The best model (i.e., XGBoost) reported AUC values ranging between 78 and 81%, AU-PRC values ranging from 51 to 69%, and Brier score ranging from 0.124 to 0.161. This study was the most comparable with ours since the authors of the study also adopted an approach based on a finite time horizon (3 to 5 days). The authors of the study similarly respected replicability and reporting guidelines [19]. While the reported results are comparable with our own (in terms of AUC and Brier score), we mention that the authors did not report the sensitivity and specificity of the models. Thus, a further comparison of the performances cannot be performed.

More in general, five recent reviews [2, 18, 27, 32, 35] surveyed the state-of-the-art with respect to prognostic ML models for COVID-19: most of the surveyed works were found to be subject to a high risk of bias. This is due to limitations related to model development and data collection [17], lack of reporting standards [4], lack of procedures to control or mitigate over-fitting, and lack of data sharing [15] which, in turn, may affect replicability.

Let us now consider our models and the performance results reported in the previous section. As a first note, the best model (i.e., the SVM) was associated with an AUC score higher than 80%, and good results for both the Brier score (i.e., calibration) and standardized net benefit (i.e., utility): indeed, SVM was the best model according to all these three metrics. All models reported good specificity (always greater than 75%), but a relatively low sensitivity (the highest sensitivity was 66%, reported by the DT model). This relative imbalance between the two performance metrics can be seen as a consequence of the observed label imbalance: indeed, since the negative class was strongly over-represented, all models learned to recognize negative instances more accurately than positive ones.

About calibration, all models reported a similar calibration pattern, as shown in Figure 5. Indeed, it can be easily seen that all models tended to be over-confident in their predictions: the mean predicted probability scores were consistently higher than the corresponding fractions of positive instances. Nonetheless, the best performing model (i.e., SVM) reported a good Brier score value.

Finally, in regard to utility, we see that the cut-off point for positive utility for the best model (i.e., SVM) is $\tau \sim 0.5$, while the utility was strictly positive for every threshold lower than that value. In particular, at the selected threshold value ($\tau = 0.1$), all models were associated with positive utility, which was greater than both the Treat-All and Treat-None models (i.e., respectively, the models that always and never predict ICU admission). This last result, in particular, shows the potential usefulness of the developed ML models in this critical setting.

While we believe that these results are promising, we also acknowledge the following limitation: the generalizability of the developed models was not externally validated, either on data collected from different settings or collected from the same hospital but in different periods. Nonetheless, as we noted in the Methods section, the adopted model development procedures were selected to increase model robustness, reduce over-fitting, and avoid any form of data leakage. Moreover, coronavirus diseases are known to be subject to relevant changes over time, and also this puts this limitation in a different perspective than it is usually the case: indeed, the robustness of any predictive model should always be assessed in light of the similarity between the training dataset and a representative sample of local and current cases, as discussed in some recent contributions (like [4, 5]). In fact, since the robustness of any ML given model would be jeopardized by the constant mutability of the SARS-CoV-2 virus, this study does prove the potential utility of ML for this clinical task, even when parsimoniousness and robustness are pursued over mere accuracy.

**Fig. 7:**
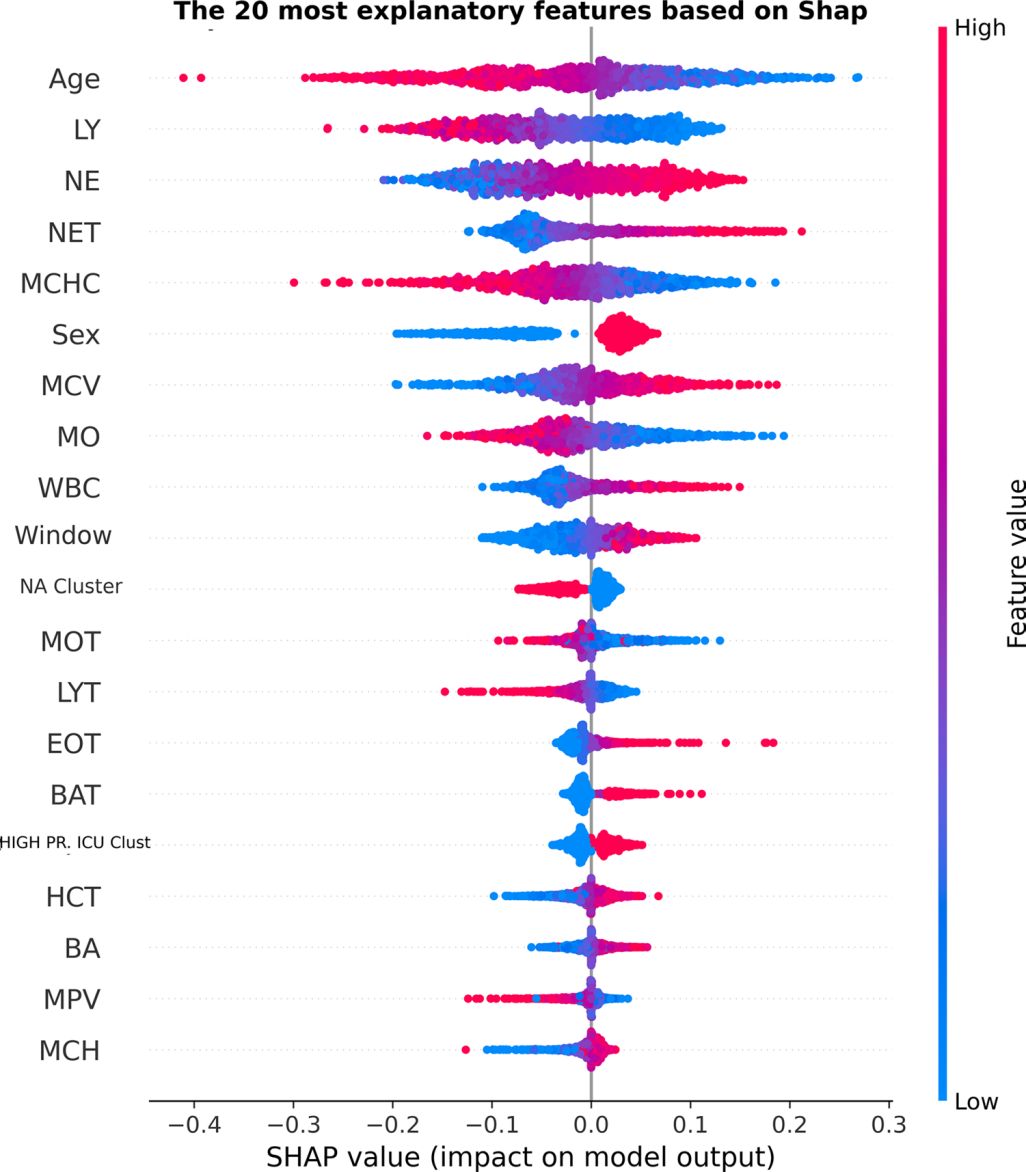
Shapley value–based interpretability analysis of the developed SVM model. For the sex variable, 1 denotes a male patient while 0 a female patient

As an additional supporting element of our study, we also performed an interpretability analysis based on the Shapley value method [24]. The results of this analysis are reported in Figure 7, and rendered in terms of feature importance attributions for the 20 most relevant features.

As depicted in Fig. 7, lymphocytes and neutrophils counts were among the most significant features. In particular, low lymphocytes values and high neutrophils values were predictive for ICU admission. This finding, which was also reported in relation to the clustering analysis Section 2.1, is consistent with several studies in the existing literature [16, 28, 29], where the neutrophils-to-lymphocyte (NLR) ratio, compared with other laboratory parameters that predict the prognosis of COVID-19, is considered as one of the most important and practical prognostic factors for clinical application, also due to its convenient derivation from routine blood tests [31]. Indeed, the neutrophil-to-lymphocyte ratio (NLR) is an inflammatory marker easily calculated by dividing absolute neutrophil count by absolute lymphocyte count, which are two parameters routinely extracted in clinical settings. Recently, studies have reported that NLR levels were higher in more severe patients and were suggested to have high prognostic value in COVID-19 patients [8]. COVID-19 severity is primarily affected by the innate inflammatory response of the body, where more severe cases were attributed to cytokine storm, a condition when there is an excessive immune response [8]. The biological mechanism underlying this association is that high NLR indicates an imbalance in the inflammatory response, which resulted from increased neutrophil and decreased lymphocyte counts. Inflammatory factors related to viral infection, such as interleukins and granulocyte colony-stimulating factors, could stimulate neutrophil production, and, at the same time accelerate lymphocyte decrease according to various molecular mechanisms [31]. Therefore, obtaining neutrophils and lymphocytes levels upon hospital admission could allow for early risk stratification, by identifying patients with higher levels of inflammation who should be prioritized as severe COVID-19 patients.

Similar points can be made in regard to the total number of white blood cells and the other components of the leukocyte formula [14, 21]. In regard to sex, the likelihood to be admitted in ICU is higher within the male group.

We note that the information about clustering and the temporal window (i.e., the number of days of stay) was also among the most relevant features. This finding should not seem surprising: in fact, the clustering features (i.e., the severity cluster and the NA cluster in Fig. 7) were purposely built in order to provide a reliable aggregate indicator of the patient health condition. In particular, through the clustering analysis reported in 2.1, a positive correlation was found between the output of the clustering procedure and the features of patients admitted to the ICU. Similarly, higher window variable values indicate a longer hospital stay, which could similarly be a sign of a more complicated disease course.

As a final remark, we note that our models were developed based on data from the first wave of COVID-19 (in Italy), that is with likely the SARS-CoV-2 Wuhan strain, and only based on ED data. It could be argued that the following waves and the related hospitalized populations were (and will be) at least partially different from the first-wave one, especially in terms of phenotype and disease bio-markers, also for a matter of vaccine-induced partial immunity against COVID-19. The most obvious element of difference regards the reduction of the age of hospitalized patients (due to the higher incidence of vaccination in the elderly) [25], but also other potential elements could be factored in. Furthermore, accounting also for non-ED patients could result in differences regarding which individuals develop critical illness, with respect to the one we observed in this study. In order to address this potential *concept drift*, in future works, we plan to externally validate our models with data coming from other hospitals and other time periods as already undertaken for the diagnostic task in [5]. This would also allow to test the model in light of possible virus variations, different patient management policies and therapeutic treatments. To this end, however, we also note that, generally speaking, the use of ML models should always be limited to settings that produce (and consume) data that are relatively similar to the training population, as shown in [5].

## Conclusions

In this paper, we presented the results of a retrospective study aimed at predicting whether a given COVID-19 patient will likely need to be transferred to the ICU during the following 5 days of their hospital stay. The results we report are supported by a large, publicly available dataset^2^ that the authors personally curated. In order to avoid biases and risks of overfitting, we adopted a development process based on recent study quality and reporting guidelines [4, 19]. The proposed methods, and in particular the best model (i.e., the SVM), exhibited promising performance, in terms of predictive power, calibration, and utility. Our approach is also parsimonious since our models are only based on two demographic features and the results of the CBC test, one of the cheapest, stablest, and fastest test in the laboratory medicine field: we believe that this is the primary strength of our approach, as this guarantees its applicability also to resource-constrained settings or developing countries [9]. An interpretability study, consistently with the existing literature, also backed up the validity of our models.

In future works, we aim to externally verify our models by using data from other hospitals and time periods. This will allow us to evaluate the model in light of external factors, like possible viral variants, the diffusion of viable alternatives in patient care and therapy strategies, or the effects of vaccines on the disease progression and diffusion. Furthermore, an additional line of further research could regard the combination of our methods with models predicting the worsening of health status over time [26] so as to provide clinicians with more information about patients’ health status and better risk stratification indications.

## Footnotes

1 https://zenodo.org/record/4081318#.X_1UDxYo-Uk

2 https://zenodo.org/record/4081318#.X_1UDxYo-Uk
